# A Korean emotion-factor dataset for extracting emotion and factors in Korean conversations

**DOI:** 10.1038/s41598-023-45386-8

**Published:** 2023-10-29

**Authors:** SoYeop Yoo, HaYoung Lee, JeIn Song, OkRan Jeong

**Affiliations:** 1 School of Computing, Gachon University, Seongnam-Si, 13120 Republic of Korea; 2 Scatter Lab, Seoul, 04766 Republic of Korea

**Keywords:** Computer science, Information technology, Scientific data, Software

## Abstract

Humans express their emotions in various ways, such as through facial expressions and voices. In particular, emotions are directly expressed or indirectly implied in the text of utterance. Research on the technology to identify emotions included in human speech and generate utterances is being conducted in conversational artificial intelligence technology. Despite the importance of recognizing the factors of previously generated emotions to generate emotion-based utterances, most of the existing datasets only provide the classification of emotions in text and utterances. In addition, in the case of Korean datasets, the classification of emotions is not diverse, and it is mainly biased toward negative emotion classification. In this paper, we propose KEmoFact, a Korean emotion-factor dataset for extracting emotion and factors in Korean conversations. We also define two tasks for the KEmoFact dataset, EFE (Emotion Factor Extraction) and EFPE (Emotion-Factor Pair Extraction), and propose baseline models for the tasks. We contribute to the study of conversational artificial intelligence, especially in Korean, one of the low-resource languages, by proposing the KEmoFact dataset and suggesting baseline models for two tasks.

## Introduction

As artificial intelligence-related technologies grow at a rapid pace, interest in research on human-centered artificial intelligence that focuses on humans is increasing^1^. The easiest field to experience artificial intelligence technology in everyday life is conversational artificial intelligence, such as chatbots. In the past, it was considered very difficult or impossible to outperform human-level gold standards in natural language-related tasks, but with the advent of pre-trained language models such as BERT^2^, and GPT^3^, research has been active, and results have exceeded gold standards in natural language-related benchmarks.

Due to these achievements, various studies are also being conducted in the field of conversational artificial intelligence^4^.

Conversation is one of the most important aspects of being an active member of society. During the conversation, you can learn a lot of knowledge and exchange emotions. Therefore, in conversation, human utterance contains not only information but also various emotions, and content that evokes emotions may appear in utterance. For conversational artificial intelligence to achieve human-level artificial intelligence, it is necessary to identify emotions that appear directly or indirectly in utterance and generate emotion-based answers accordingly. At this time, recognizing the factor of emotion is very important because it can help generate emotion-based answers.

However, the existing emotion dataset or emotion-based conversation dataset is concentrated on classifying emotions in text or speech. In addition, since emotional conversation systems are based on existing datasets, research on finding and utilizing the factor of emotions is very insufficient. In particular, in the case of Korean, most emotion classification datasets have limited categories of emotions or are mainly biased toward negative emotions, and there are no datasets with both emotions and factors of emotions annotated.

In this paper, we propose a Korean Emotion-Factor dataset, KEmoFact, in which emotions and factors are tagged for Korean conversations, and also propose a model that can be the baseline of the dataset^5^. We express emotion-factor rather than emotion-cause because we find out and annotate the causes and targets of emotion or all factors related to emotion. Recently, conversational artificial intelligence research using Korean has become active, and various datasets are being released. However, there is no dataset that can find emotions and factors of those emotions, and we contribute to the study of Korean-based conversational artificial intelligence by opening the KEmoFact dataset to the public. We construct a KEmoFact dataset by using the train set of existing publicly available EmpathicDialogues datasets^6^ to translate them into Korean and then annotate phrases that are the cause or target of a given emotion or are related to the emotion.

Based on this new dataset, we implement a baseline model of the Korean emotion-factor extraction task, which finds the corresponding factors when the emotion for the conversation is given as input. However, the emotion-factor extraction task has the disadvantage of requiring emotional annotation even during testing, limiting the applicability of the model. Therefore, based on the above task, we also implement the emotion-factor pair extraction task with the proposed dataset, KEmoFact, which is a task that finds emotions and factors together in conversation as pairs. These two tasks are implemented by applying them to the Korean pre-trained language model, and the experiment results are compared and analyzed.

In this paper, our major contributions are as follows: (1) We build and provide the KEmoFact dataset, a Korean emotionfactor dataset containing Korean text, emotion, and factors that include all the causes or the target of emotion. (2) We provide baseline models that allow inferring factors for a given emotion and emotion-factor pairs in Korean conversations by applying the pre-trained language model to KEmoFact datasets. (3) We contribute to research in the field of Korean-based conversational artificial intelligence by providing sufficient analysis of the KEmoFact dataset and its baseline model.

## Related work

### ECE and ECPE task

In order to implement conversational AI at the human level, the emotion-based conversation must be continued, and what causes emotion must be found and used to create conversations. Therefore, the emotion-cause extraction (ECE) task, which finds the cause when emotion is given, is being studied as an important task in the field of natural language processing. Accordingly, based on deep learning, a method of effectively extracting cause by methods such as multi-kernel SVM^7^, question-answering^8^, LSTM and SVM^9^, joint-learning^10^, co-attention^11^, and RNN-Transformer Hierarchical Network^12^ have been studied.

However, ECE tasks limit the applicability of the model because there is a limitation that emotion annotation is required even during testing. Therefore, the application of the emotion-cause pair extraction (ECPE) task, which extracts pairs of emotions and causes without emotion annotation, is required^13^. Accordingly, studies on ECPE tasks are being conducted in multi-task learning^14^, transition-based directed graph construction^15^, and inter-cause modeling^16^, etc. Based on the extracted emotion-cause relationship, potentially important information can be obtained, and based on this, empathetic dialog modeling has been used as agents such as blender-bot^17^ and persona^18^.

### Datasets on emotion

However, the number of open datasets tagging the causes of emotions along with the types of emotions is quite small, and only a few are in Korean. Using EmpatheticDialogues^6^, an emotional dataset composed of large-scale conversations released, attempts have recently been made for ECPE tasks such as releasing the EmoCause dataset^19^ by annotating emotional causes. Since EmoCause was conducted using only the validation and test set among the EmpatheticDialogues dataset, the number of data is quite small, about 5K. As such, due to the complexity of the annotation process, the size of the published datasets for ECPE is usually small. In addition, the causes of several existing datasets are mainly biased toward the target or cause of the emotion. Therefore, it is necessary to deal with the factors that have aroused emotions in a wider category.

Also, since the EmoCause dataset is in English and the label type is composed of words, there are several problems in performing ECPE in Korean based on this dataset. In English, the unit of spacing is a word, but in Korean, the unit of spacing is a clause. In general, one word forms one clause, but sometimes, a postposition is attached to a substantive, or a suffix is attached to a stem to construct a clause. In addition, since there is a linguistic feature that English and Korean have different word orders, it is impossible to apply ECPE tasks through simple translation work. Therefore, in this paper, we propose KEmoFact, an emotion-factor dataset customized for Korean with approximately 16.5K of data using the train set among the EmpatheticDialogues dataset.

## Methods

### Task description

In the conversation that people exchange, information and emotions are embedded together. For smooth conversation, it is necessary to understand the emotions, which can be called the overall atmosphere. Furthermore, it is necessary to be able to identify the factor that caused the emotion among various information. Therefore, it is an important task for conversational AI to identify the factor that caused it, along with emotions, to achieve human-level AI.

Accordingly, several studies, such as emotion-cause extraction and emotion-cause pair extraction, are being conducted. However, for the factor that aroused the emotion to be extracted, several studies have confused the words ‘cause’ and ‘factor’. The emotional cause extracted in most studies is biased toward the object, which is the person or thing that caused the emotion. However, there are not only objects but also various things, such as specific situations and causes, in order to evoke emotions. Therefore, the factor of emotion may be simply represented in one word or phrase, but the entire sentence may be a factor of that emotion. In other words, it can be seen that the factor is recognized as a wider category than the cause and is covered comprehensively.

In our proposed Task, we use the term "factor" rather than "cause". For clarity, we define the meaning of ’cause’ and ’factor’ as defined by the Cambridge Dictionary^20^ in Table 1. Many factors can make up a cause. No single cause is responsible for an emotion, but rather, multiple factors can impact an emotion. Therefore, we want to focus on the many factors that lead up to an emotion rather than a single cause.

**Table 1:** Definition for emotion and factor.

Word	Definition
Emotion (noun)	The reason why something, especially something bad, happens
Factor (noun)	A fact or situation that influences the result of something

Finally, we define the name of the dataset that we propose as KEmoFact, a Korean Emotion-Factor dataset, and we extend the ECE task and define it as an emotion-factor extraction (EFE) task. Furthermore, a task that extracts emotion and cause as a pair from the conversation without being given emotional annotation is defined as an emotion-factor pair extraction (EFPE) task.

## KEmoFact dataset

We propose a dataset, KEmoFact, in which the factors of emotions are annotated in the Korean text that could infer the factors of emotions or emotions in Korean utterances. This section summarizes what data was utilized to build the KEmoFact dataset, how the annotation was conducted, and finally, the analysis results for the dataset.

### Data collection

In this paper, we utilize the EmpathicDialogues dataset^6^ released by Facebook research to help the dialog agent understand the emotions of the human and learn to provide the appropriate conversation for that emotion. This dataset is an open-domain conversation that allows conversations on various topics, with speakers and listeners in one-on-one conversations. The speaker starts a conversation about his or her overall situation and feelings, and the listener provides a response by considering the other person’s feelings, expressing empathy, and exchanging more than six turns, which consists of a total of about 25k conversations.

Each conversation contains one specific emotion among 32 emotions except for neutrality, and each emotion is evenly distributed, allowing detailed emotion classification in each conversation through the dataset, thus generating a conversation that expresses emotion. Therefore, in order to proceed with the EFE and EFPE task in Korean, we intend to construct the KEmoFact dataset by annotating the factor of emotion using the ‘prompt’ that is more likely to contain emotional factors (than a general conversation) because it represents the overall situation of the conversation and the corresponding ‘emotion’ column in the EmpatheticDialogues dataset.

### Data annotation

The Annotation task can be largely divided into two stages. First, we translate the conversation data and emotion label of EmpathicDialogues^6^ from English into Korean. Next, we annotate the factors that evoked emotion in the conversation in Korean. We annotate using the EmpathicDialoges dataset’s train set, which consists of 19,533 conversations. However, a total of 17,798 conversation data are used except for missing values such as no emotion or prompt. So, 10 Koreans who are fluent in Korean as their native language participate as annotators to build a KEmoFact dataset considering context, emotion, and the factors of emotion. Seven people, including the author of the paper, conducted an annotation of 2,000 examples and the remaining three, about 1,300 examples. Furthermore, in the process of tagging the emotional factor, in order to increase the quality of the dataset by excluding individual subjectivity, the review work was also carried out, as many annotations were conducted on data that were not conducted by the person.

• Step 1. Translation

Python library googletrans^21^ and Pororo^22^ library are used together to translate the text ‘prompt’ of the EmpathicDialogues dataset into Korean. The googletrans is a library that implemented Google Translate API. Both Pororo and Google Translate API^23^ are neural network-based translation models, and the annotators compare the two results and select more appropriate results according to the context of the sentence. If both results translated into Korean are awkward or incorrect, the corresponding instance is excluded from the data.

However, the 32 emotions used in the EmpathicDialogues dataset contain emotions that are very similar to each other. In particular, some emotions are very similar in Korea and are difficult to distinguish. We perform the process of combining emotions that appear as synonyms in the Cambridge Dictionary^20^ among 32 emotions into one emotion in order to classify them as fully understandable emotions in Korean definitions. Table 2 shows the newly categorized emotion labels. We integrate ’annoyed’ and ’furious’ into ’angry’. ’anxious’, ’apprehensive’, and ’terrified’ are integrated into ’afraid’. ’ashamed’ and ’guilty’ are also integrated into ’embarrassed’. In addition, we change ’devastated’ to ’disappointed’, ’faithful’ to ’trusting’, and ’nostalgic’ to ’sentimental’. Finally, we use 22 emotions as emotion classification for datasets: afraid, angry, anticipating, caring, confident, content, disappointed, disgusted, embarrassed, excited, grateful, hopeful, impressed, jealous, joyful, lonely, prepared, proud, sad, sentimental, surprised, and trusting.

**Table 2:** Newly categorized emotion labels.

Emotion	Definition	Korean	New emotion
Afraid	To feel fear or worry	무서워하는	Afraid
Anxious	Worried and very nervous	불안한	
Apprehensive	Feeling anxious about something that you are going to do	불안해하는	
Terrified	Very frightened	무서워하는	
Angry	Feeling that you want to shout at someone or hurt them because they have done something bad	화난	Angry
Annoyed	A little angry	짜증난	
Furious	Very angry	매우화가난	
Ashamed	Feeling angry and disappointed about someone or something, or because you have done something wrong feeling ashamed or shy	부끄러운	Embarrassed
Embarrassed		당황한	
Guilty	Feeling bad because you have done something wrong	죄책감이드는	
Devastated	Feel very shocked and upset	충격을받은	
Disappointed	Sad because something is not as good as you expected, or because something did not happen	실망한	Disappointed
Faithful	Always liking and supporting someone or something to believe that someone is good and honest and will not harm you	충실한	Trusting
Trusting		믿는	
Nostalgic	Feeling both happy and sad when you think about things that happened in the past related to feelings and memories and not related to how much money something costs	향수에젖은	Sentimental
Sentimental		감정적인	

• Step 2. Annotation

Emotion cause is the factor that has been studied in emotion processing along with emotion classification. Although a direct cause, such as any event or object that triggers an emotion, can provide important information through interaction with the emotion, emotions are related to many other factors besides the cause. Therefore, we would like to find factors related to emotions that can consider not only the cause or targets of emotions but also the situation of emotions in conversation.

We recruited 10 annotators to annotate the KEmoFact dataset. All 10 annotators are native Korean speakers who were born in Korea, have lived there for more than 20 years, and are either attending or have graduated from university, making them very familiar with the Korean language and culture. The 10 annotators were not paid for their annotations but volunteered to participate and follow the guidelines provided by the authors. The entire dataset was distributed among 10 annotators, one annotating each example. To ensure dataset quality, the entire dataset was reviewed by three of the authors for annotation results, with at least two of the authors agreeing on the data.

10 annotators annotate an emotional factor from each context according to some basic rules. First, since the conversation is in Korean, the factor is selected in terms of phrase and tagged based on spacing. Thus, the factor of emotion can be one or several clauses that make up a sentence, but it can be an entire sentence. In addition, multiple selections are possible because there may be more than one factor that causes emotions in each conversation. If multiple selections are made, connect factors using ‘###’ for the delimiter.

Here, there are some cases that can cause confusion in learning by providing shortcuts to models that extract factors from sentences if they contain direct emotions in the factor of emotions or if they are located directly before or after emotions. Therefore, in these cases, we select the phrase of the factor, excluding direct emotional expression. Additionally, the instances are excluded if there are too few or ambiguous hints about the factor in the context or if the context is composed only of words and is too short.

### Data analysis

The KEmoFact dataset contains a total of 16,532 data, and the train, valid, and test sets are divided at a ratio of 8:1:1, respectively. The dataset is randomly divided with the same emotion distribution maintained similarly by the existing EmpathicDialogue^6^ dataset. We leverage the Stratified KFold library^24^ provided by Scikit-learn to divide datasets while maintaining distribution.

**Figure 1:**
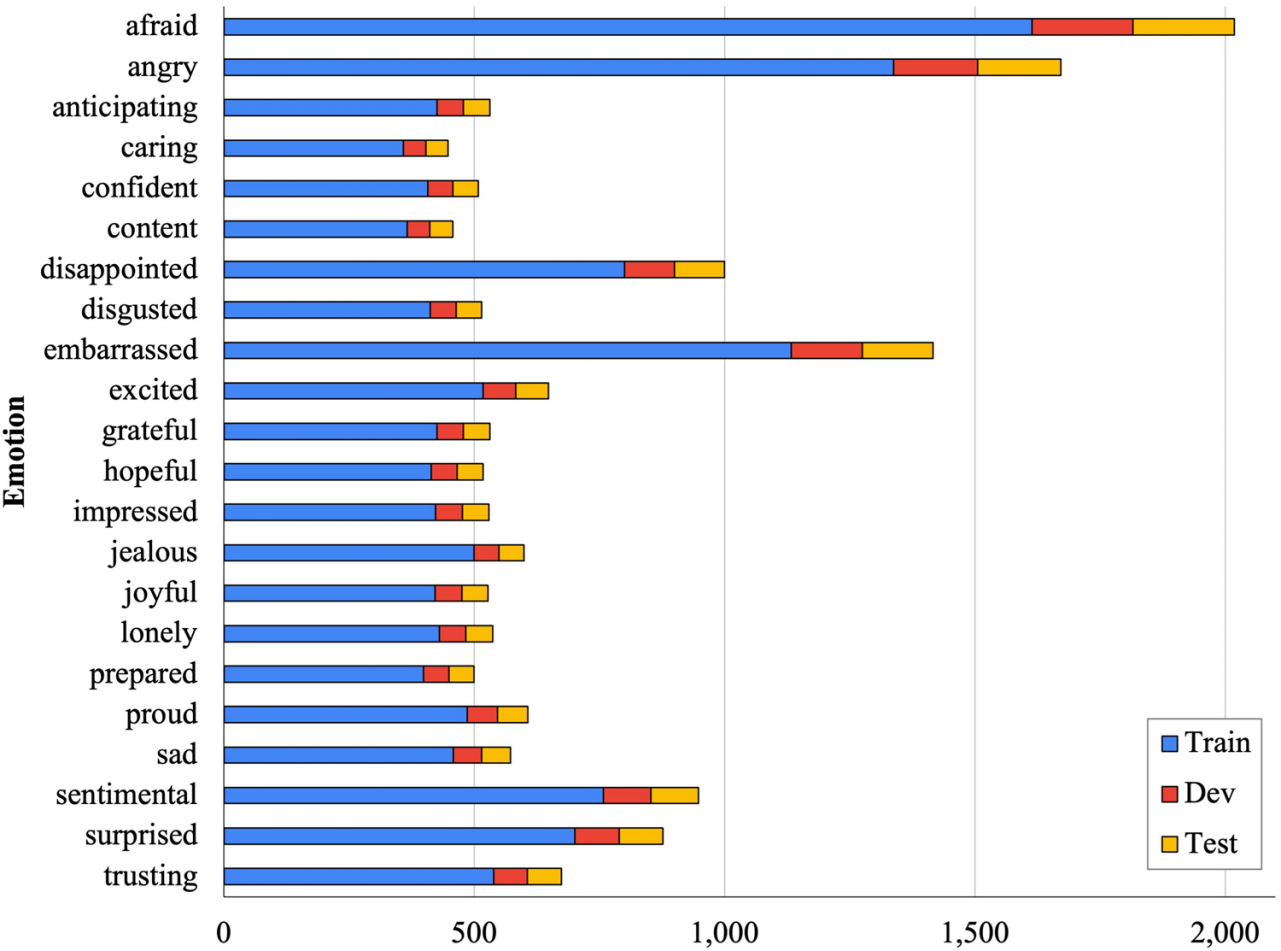
Statistics of KEmoFact dataset.

Figure 1 shows the statistics of the KEmoFact dataset for each emotion. The KEmoFact dataset has 13,225, 1,653, and 1,654 instances for train, dev, and test set, respectively. The dataset has 22 emotions, and instances are distributed for each emotion. In the process of resetting the existing 32 emotions into 22 emotions, emotions such as ’afraid’, ’angry’, and ’embarrassed’ have relatively more instances than other emotions, and other emotions show a similar size of instances.

Table 3 shows some examples of the KEmoFact dataset. We take ‘conv_id’ and ‘emotion’ from the EmpatheticDialogues dataset for future use. In the EmpatheticDialogues dataset, the ‘prompt’ column, which is used as a column explaining the situation of conversation, is changed to a column named ‘context’ in KEmoFact, and the Korean-translated text is added as the ‘kor_context’ column. The part corresponding to the factor of emotions that annotators directly annotated is added as a ‘factor’ column, and since it can contain more than one phrase, we use ‘###’ as a separator token for the multiple factors. There are 15,246 instances containing only one phrase, accounting for about 92% of the total data, and 1,286 instances with two or more phrases, accounting for about 8%.

**Table 3:**
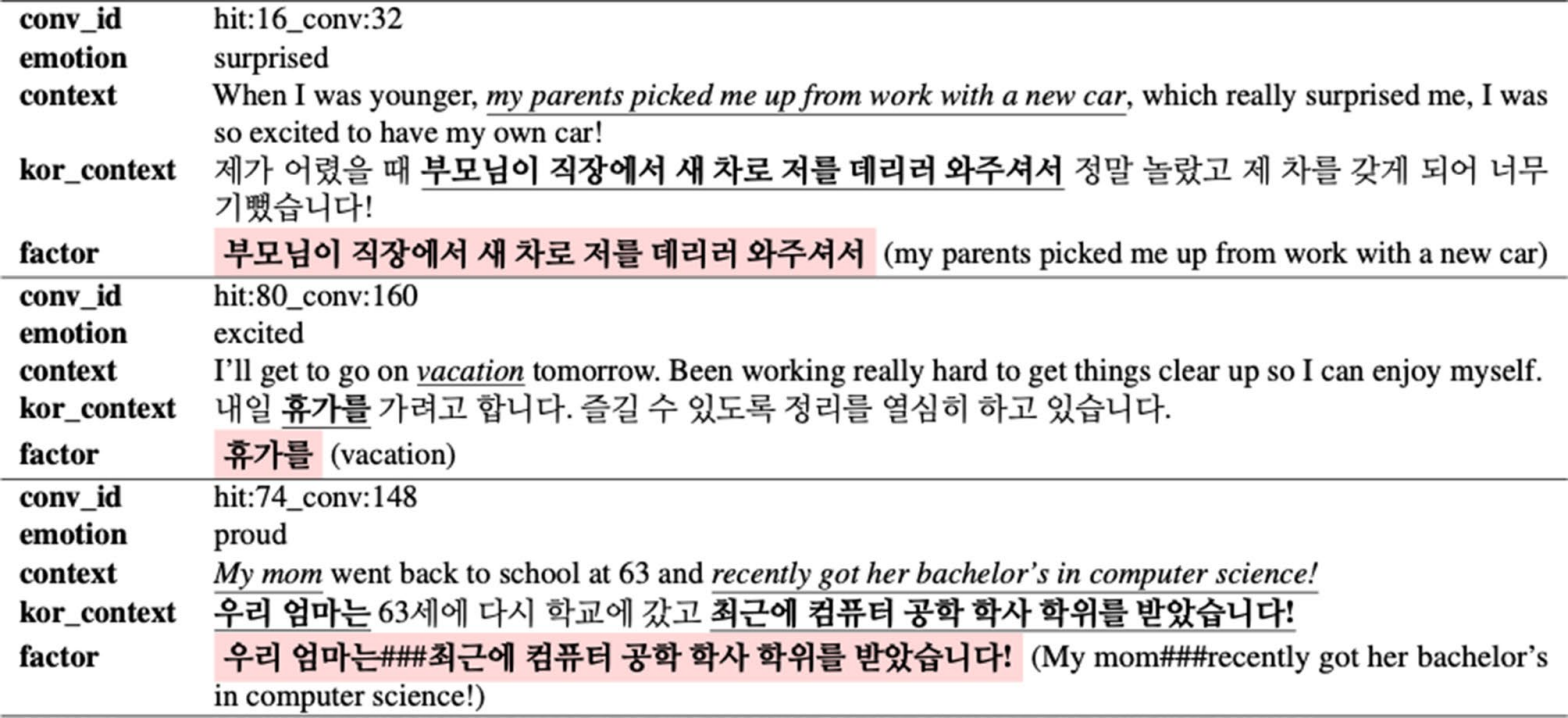
Examples of KEmoFact dataset.

## Proposed model

Using the proposed KEmoFact dataset, we propose baseline models for the emotion factor extraction task, which extracts the factors of emotion when given emotion, and the emotion-factor pair extraction task, which extracts both emotion and factor as a pair from Korean conversations.

### Emotion factor extraction (EFE) task

We preprocess the KEmoFact dataset according to the EFE task and apply the published Korean pre-trained language model. We choose the token classification approach to solve the problem, like a kind of named entity recognition (NER) task, to extract the factor of emotion when given emotions and text. We use token classification to extract multiple factors within the text because more than one factor may appear in the text. To solve the problem with token classification, we use BIO tagging^25^, which is commonly used in the NER task. The BIO format uses I-prefix, B-prefix, and O tags. By applying this method to our model, each token is represented by B-FACTOR, I-FACTOR, and O tags. B-FACTOR represents the token from which the factor begins, I-FACTOR represents the token inside the factor, and the O tag represents the token outside the factor.

**Figure 2:**
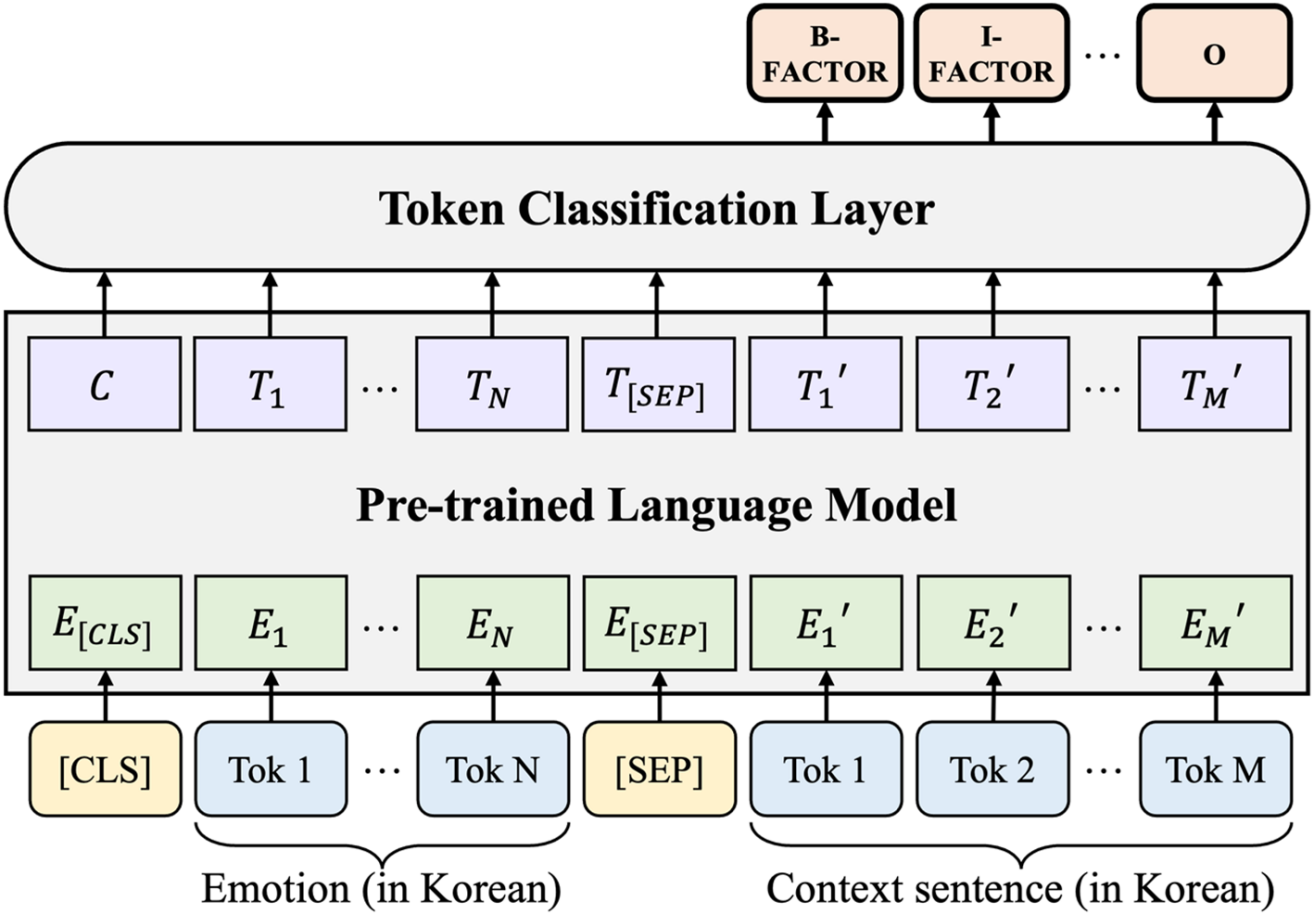
Baseline model architecture for EFE task.

Figure 2 shows the structure of our proposed baseline model. For the learning of the model, we connect the emotion translated into Korean and the context sentence translated into Korean with the [SEP] token and use it as input for the pre-trained language model. Rather than using the existing emotion words in English as the dataset has, they are translated into Korean and entered into emotion so that the Korean language model can understand them a little better. Finally, through the token classification layer classified by B-FACTOR, I-FACTOR, and O tags, the tag with the highest probability value is shown as output.

### Emotion factor pair extraction (EFPE) task

We also propose an EFPE task as a baseline model that can overcome the shortcomings of the EFE task that can only be performed if emotions are essentially given. Same as the task above, we preprocess the KEmoFact dataset and apply it to the published Korean pre-trained language model to perform token classification. However, unlike the EFE task, emotion is not given as input, so the pre-processing process is carried out by applying BIO tagging to the emotion like the factor. Therefore, it is applied according to each emotion name according to the emotion classification of the KEmoFact dataset, and for example, if the emotion is afraid, it is expressed as B-afraid, I-afraid, and O tags.

**Figure 3:**
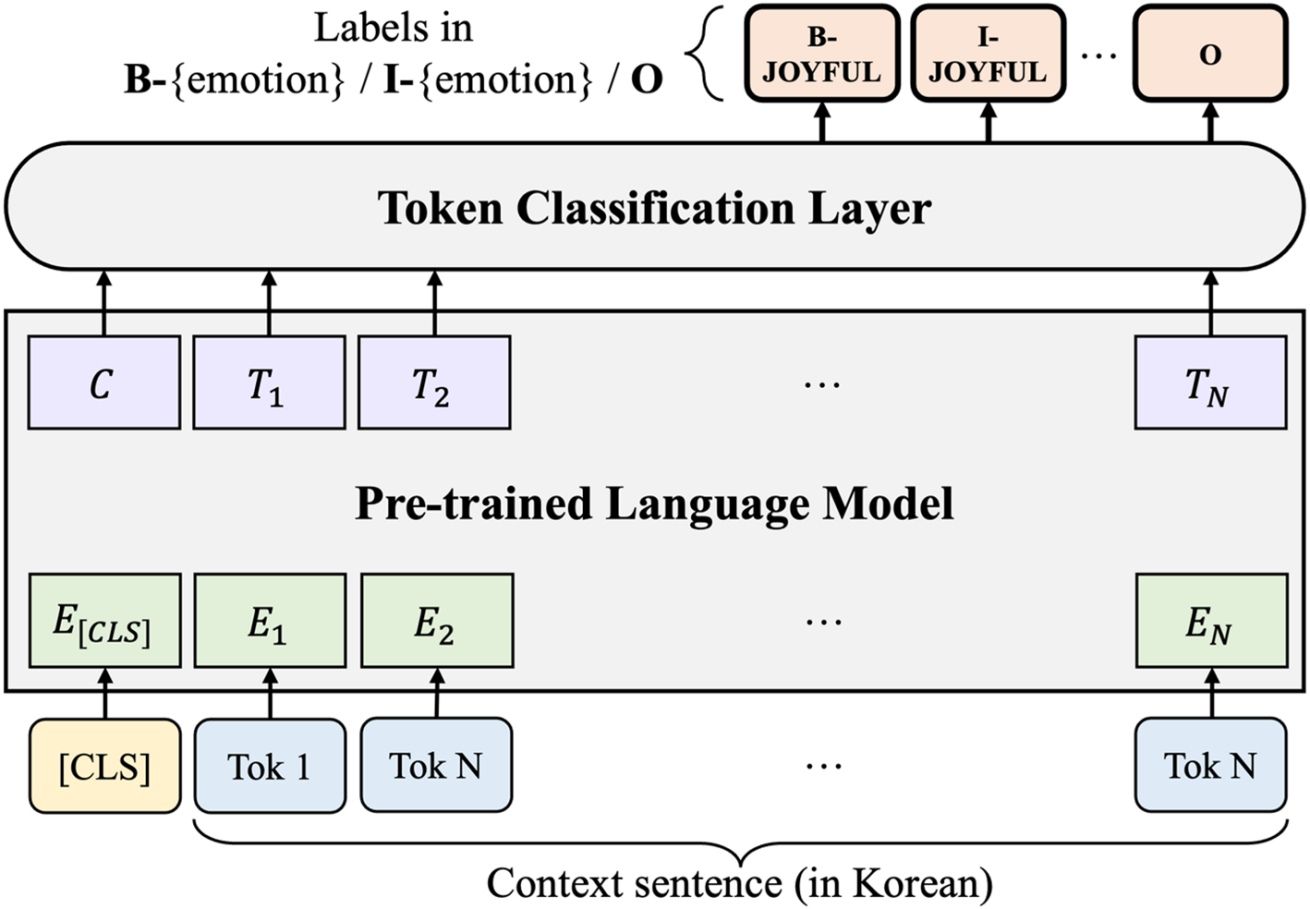
Baseline model architecture for EFPE task.

Figure 3 shows the architecture of the baseline model for the EFPE task. To perform the EFPE task, the model receives only sentences as input. The model classifies each token as the most appropriate tag among B-emotion, I-emotion, and O tags from the input sentence. Because it classifies using tags for all emotions, it is possible to extract multiple emotion-factor pairs from one input through post-processing.

## Results

In this paper, we implement a model that extracts the factors of emotion and emotion-factor pairs from Korean dialogues using the KEmoFact dataset. To this end, we apply the three published Korean pre-trained language models and then propose the baseline model by comparing the experiment results.

### Experimental setup and metrics

We utilize Google Colab and a Tesla T4 16GB GPU for the experiment. We set max_sequence_length, which means the number of input tokens of the model, as 128 according to the distribution of the total number of tokens of the KEmoFact data. For the comparative experiment by model, the learning rate is 5e-5, the batch size is 32, and the epoch is 5, all of which are equally applied. Also, we use an Adam optimizer and a linear type of learning rate scheduler.

We use precision, recall, F1 score, and Jaccard score as metrics for experiments. Since our proposed model utilizes the token classification method, we calculate precision, recall, and F1 score using the seqeval^26^ module provided by Huggingface, a metric module mainly applied to token classification tasks such as named entity recognition. However, there is a limit to evaluating the model performance for the task we propose using only the metric applied by the existing token classification task. We use word-level Jaccard score^27^ as a metric because it is more important for model evaluation to determine how much the predicted factor is included in the actual sentence. The Jaccard similarity coefficient measured how similar the predicted results from ground truth are. The equation of the Jaccard similarity is as follows: *J*(*G,P*)= |*G*∩*P*|*/*|*G*∪*P*|, where *G* represents the set of words in the gold standard sentence, and *P* represents the set of words in the predicted sentence.

### Baseline models

We select the final baseline model with the proposed method and conduct a comparison experiment by selecting three Korean pre-trained models to validate it: DistilKoBERT^28^, KoElectra-base^29^, and KcElectra-base^30^. Although there are pre-trained language models that support multiple languages, such as BERT and Electra, we select the Korean models that perform better than the existing multilingual models on several Korean benchmarks.

DistilKoBERT^28^ model is a lightweight version of KoBERT^31^ that has learned the BERT^2^ model in Korean. It trained on about 10GB of data, including Korean wikis, Namu wikis, and news. It has a size of 108MB compared to 681MB for the multilingual BERT model and shows relatively good or similar performance for Korean subtasks.

KoELECTRA^29^ model This model is trained on 34GB of Korean data, including data from wikis, tree wikis, newspapers, messengers, and more, using the ELECTRA^32^ model. There is a large model and a base model, and both models show better performance than DistilKoBERT on Korean benchmarks.

The KcELECTRA^29^ model is a model that trained the ELECTRA^32^ model with about 45GB of Korean data, just like the KoELECTRA model. The main difference is the data used for training. Most of the published Korean Transformer series models are trained on well-refined data such as Korean wikis, news articles, and books. However, the data used in practice is unrefined, and colloquial features include many neologisms, typos, and other expressions that do not appear in formal writing. To apply KcELECTRA to a dataset with these characteristics, the authors collect comments and replies from online news and train it. It shows better performance than DistilKoBERT and KoELECTRA on Korean benchmarks.

### Experiment results

**Figure 4:**
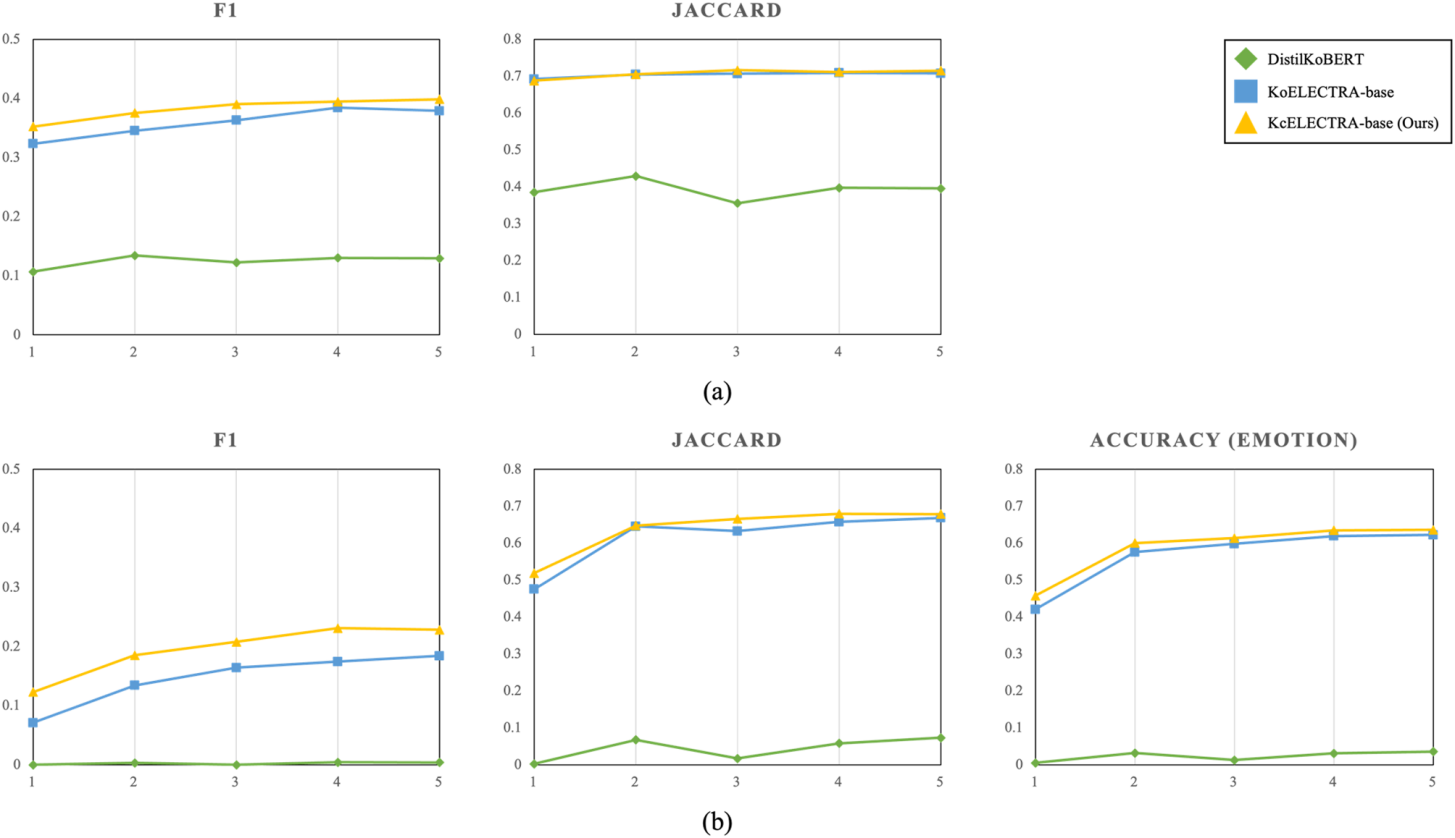
Results of models over epochs.

Using the KEmoFact dataset, we experiment and compare the performance of the models for two tasks, the EFE and EFPE tasks. First, we run a comparison experiment for each epoch to select the best-performing model as the final baseline model. Figure 4 graphically shows the results of the experiment. (a) shows F1 and Jaccard scores as results for the EFE task. (b) The results for the EFPE task show F1, Jaccard score, and accuracy for emotion classification. For both tasks, the best results are obtained using the KcElectra model, which is a good fit for KEmoFact’s conversational dataset because it contains relatively more colloquial data, such as comments.

Table 4 shows the results of experiments using DistilKoBERT, KoELECTRA-base, and KcELECTRA-base models for each task. The table shows the experimental results of the EFE task and EFPE task separately, and in the case of the EFPE task, the accuracy of emotion classification is also shown because emotion and factor are extracted as a pair from a given sentence. As shown in Table 4, the KCELECTRA-base-based model shows the best performance in both tasks. We propose a KCELECTRA-base-based model as the final baseline model of the KEmoFact dataset.

**Table 4:** Experiment results on DistilKoBERT, KoELECTRA-base, and KcELECTRA-base.

Task	Model	Loss	Precision	Recall	F1	Jaccard	Accuracy (Emotion)
EFE Task	DistilKoBERT	0.6353	0.1922	0.1524	0.1700	0.4371	-
	KoELECTRA-base	0.5635	0.3754	0.4417	0.4058	0.7349	-
	KcELECTRA-base	0.5879	0.4106	0.4519	0.4302	0.7450	-
EFPE Task	DistilKoBERT	1.7166	0.0137	0.0022	0.0039	0.0916	0.0345
	KoELECTRA-base	1.0290	0.1739	0.2415	0.2022	0.6892	0.6197
	KcELECTRA-base	1.0671	0.2272	0.2813	0.2514	0.7016	0.6433

When the performance is calculated with the F1 score, it can be seen that the score is achieving a relatively low score compared to the performance of the recent NER task. Since the F1 metric used in this experiment uses the method applied to the token classification method as it is, it is based on whether each predicted token matches the same label as ground truth. However, because we need to find a factor within a given sentence, it is important to how similarly we infer from the actual ground truth, so the Jaccard similarity score is a more suitable metric for our task.

In particular, in the EFPE task, the F1 score shows relatively low results. The EFPE task is more complex than the EFE task because the EFPE task requires the extraction of emotions and factors in pairs only from input sentences. In addition, the model we propose seems to have low scores on the F1 score because our model can extract multiple emotion-factor pairs in one instance, but the instance in the current KEmoFact dataset is classified as only one emotion. However, as shown in the accuracy of the emotion classification and Jaccard similarity score, the proper inference is possible in our proposed model even though it is a challenging task.

Table 5 shows the results by sentiment. It shows results for the EFE task and EFPE task by emotion using our final baseline model, the KcElectra model and shows the key metrics F1, Jaccard, and Accuracy for each emotion. The best-performing emotions are different for each task and metric. In the EFE task, *excited* and *jealous* are the top performers, while in the EFPE task, *angry*, *jealous*, and *sentimental* are the top performers. Not only does *jealous* have the highest Jaccard score in both tasks, but overall, the Jaccard scores show a similar distribution of scores in emotions. It shows that the additional Jaccard score for the KEmoFact task is a meaningful metric.

**Table 5:** Experiment results on Emotions.

Emotion	EFE Task		EFPE Task		
	F1	Jaccard	F1	Jaccard	Accuracy (Emotion)
Afraid	0.4298	0.7036	0.3099	0.6579	0.7673
Angry	0.4279	0.7923	0.3278	0.7584	0.7246
Anticipating	0.4779	0.7336	0.2833	0.7229	0.4528
Caring	0.3529	0.6154	0.1538	0.4853	0.5778
Confident	0.3158	0.6001	0.1176	0.5995	0.5490
Content	0.4000	0.7712	0.2281	0.7028	0.6304
Disappointed	0.4737	0.8041	0.2372	0.7677	0.5400
Disgusted	0.3471	0.8001	0.2373	0.7768	0.7255
Embarrassed	0.3792	0.7567	0.2733	0.7300	0.7518
Excited	0.5369	0.7818	0.0909	0.7276	0.4000
Grateful	0.3548	0.7233	0.2362	0.6542	0.6792
Hopeful	0.3846	0.6884	0.1791	0.6130	0.5577
Jealous	0.4915	0.8690	0.3065	0.8167	0.7200
Joyful	0.5357	0.7923	0.2167	0.7718	0.4038
Lonely	0.5345	0.7354	0.3200	0.7146	0.7407
Prepared	0.4587	0.6839	0.2479	0.6487	0.6600
Proud	0.3972	0.7756	0.2394	0.7732	0.6066
Sad	0.5120	0.7989	0.1831	0.7416	0.4211
Sentimental	0.4811	0.7870	0.3167	0.7455	0.7895
Surprised	0.3200	0.6948	0.2587	0.6963	0.6477
Trusting	0.3234	0.5179	0.1317	0.4390	0.5441

## Discussion

Table 6 shows an example of a case in which the model does not answer correctly. Like the first case, there are cases where the Korean postpositions or demonstrative pronouns could not be selected because the sentence is classified at the token level for learning the model. In addition, Korean postpositions or demonstrative pronouns that typically appear frequently in all sentences are a confusing factor in the model’s learning and prediction, which can be a deduction factor for experiment metrics. Like the second case, ground truth is tagged several phrases briefly, but the model sometimes predicts as a single sentence, so it is judged that it is not the correct answer. As an example, it may appear that the model performance is low, but we can see that the model is predicting quite well as we intended.

**Table 6:**
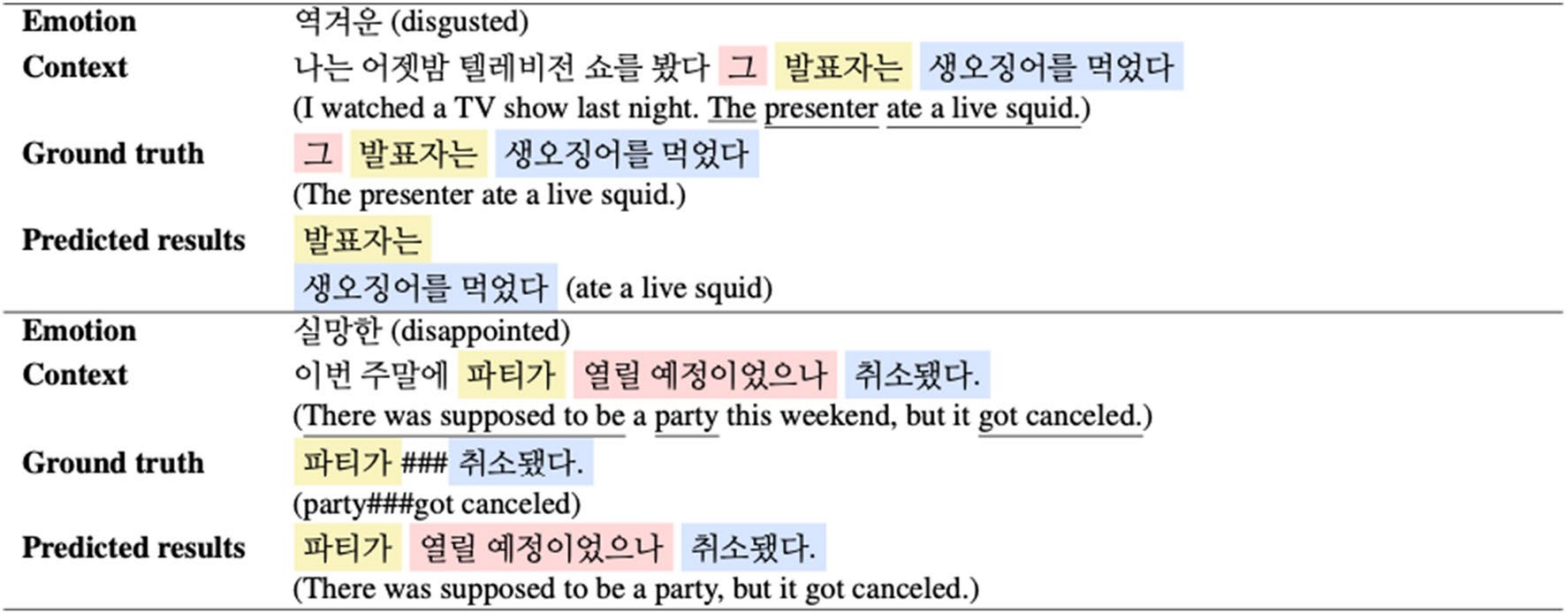
Examples of wrong answers.

We also check the results by changing emotions to see if our model can find the factors of a given emotion well when emotion and text are given. To this end, we confirm that the results predicted by the model vary as different emotional conditions are delivered in sentences containing multiple emotions.

Table 7 shows the results predicted by the model according to the given emotions in a sentence containing several emotions. The result value predicted by the model varies when two different emotions are entered into the model by creating the same sentence with a mixture of different emotions. In particular, in the first case, when very conflicting emotions of ‘trusting’ and ‘disgusted’ were given, the predicted factors fit well depending on the emotion. Of course, in some cases, the model does not work well depending on emotions, but it could be solved by making a robust model by supplementing the context in which various emotions are mixed in the learning data.

**Table 7:**
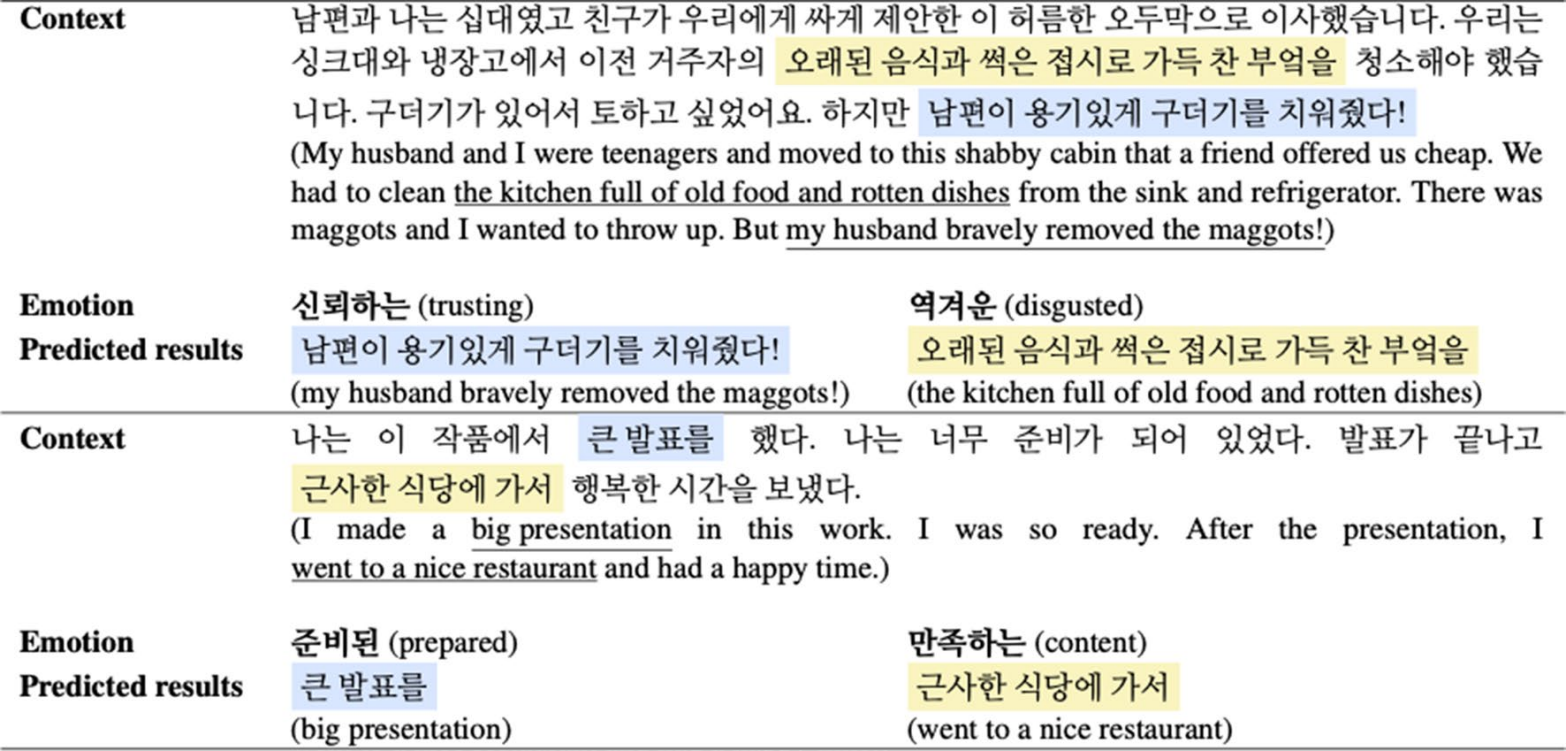
The results predicted by the model based on the emotions given in a sentence containing multiple emotions.

## Limitations

We propose the dataset for emotional factors for the lack of datasets that include both emotions and emotional factors, especially in Korean. In addition, we build and propose baseline models for extracting the factor of emotions and pairs of emotion-factor from Korean conversations using a new KEmoFact dataset so that it can be used for various studies based on emotions and factors in the future. However, there are still some limitations, so we would like to discuss them.

First, is the sentence translated into Korean appropriate? There are unnatural parts because ‘kor_context’, which is used as a Korean sentence to predict factors, is basically a sentence that translates English text into Korean. Korean has many differences from English in word order and form. Both Pororoy^22^ and Google Translation API, which we used for translation, show good performance with translation models based on neural networks, but there are still some cases where they show inappropriate results. We could improve the quality of the KEmoFact dataset using an improved translation model or utilizing sentences made in Korean from the beginning.

Second, is emotion annotation appropriate? Since the KEmoFact dataset is based on the existing EmpathicDialogues dataset, emotion annotation also follows its classification. The EmpathicDialogues dataset has 32 emotions, the criteria for which are unclear, and some emotions have ambiguous or overly detailed criteria. Although we reclassified similar emotions and organized them into 22 emotions, ambiguity about the emotion classification criteria may still exist. In particular, since there may be differences in emotional standards according to language and culture, reclassification may be necessary as a standard for emotion classification suitable for Korean. After clarifying the criteria for emotion classification, the reclassification work according to the new criteria can also affect the quality of the KEmoFact dataset and the performance of the emotion and emotion-factor extraction model.

## Conclusion

Emotion is a very important element in human conversation. In order for conversational artificial intelligence to develop into human-centered artificial intelligence, emotion-based conversation must be possible. To this end, it is important to identify the factors of emotions as well as extract emotions from conversations. However, in the past, there were datasets only for emotion extraction, and in particular, there were no datasets with emotions and factors annotated for Korean. We construct and provide the KEmoFact dataset, a dataset containing Korean context, emotions corresponding to the context, and factors of the emotions. In addition, we define two tasks for the KEmoFact dataset, EFE(Emotion Factor Extraction) and EFPE(Emotion-Factor Pair Extraction) task, and then implement baseline models that can extract the factors of emotion and pairs of emotion-factor from Korean conversations by utilizing the KEmoFact dataset.

Our proposed KEmoFact dataset and baseline models could be utilized for various Korean conversational artificial intelligence studies in the future. In particular, it is possible to provide more satisfactory answers to humans by generating appropriate utterances based on the extracted factors of emotions. To improve the KEmoFact dataset, we could find out not only the causes of the emotion but also the factors and temporal information of the emotion in a wider category. Our results confirm the value of the KEmoFact dataset for conversational AI research on Korean and further similar low-resource languages.

### Supplementary Information

Supplementary Information 1.

Supplementary Information 2.

Supplementary Information 3.

## Data Availability

All data generated or analyzed during this study are included in this published article and its supplementary information files.
